# GENI: A web server to identify gene set enrichments in tumor samples

**DOI:** 10.1016/j.csbj.2023.10.053

**Published:** 2023-10-31

**Authors:** Arata Hayashi, Shmuel Ruppo, Elisheva E. Heilbrun, Chiara Mazzoni, Sheera Adar, Moran Yassour, Areej Abu Rmaileh, Yoav D. Shaul

**Affiliations:** a Department of Biochemistry and Molecular Biology, The Institute for Medical Research Israel-Canada, Faculty of Medicine, Hebrew University of Jerusalem, Jerusalem 9112001, Israel; b Info-CORE, Bioinformatics Unit of the I-CORE at the Hebrew University of Jerusalem, Jerusalem, Israel; c Department of Microbiology and Molecular Genetics, The Institute for Medical Research Israel-Canada, Faculty of Medicine, Hebrew University of Jerusalem, Jerusalem 9112001, Israel; d School of Computer Science & Engineering, The Hebrew University of Jerusalem, Jerusalem, Israel

**Keywords:** Bioinformatics, Cancer biology, TCGA, Gene Set Enrichment Analysis, Web-based tools, Clinical data, Tumor samples, Cancer-associated molecular mechanisms, Multi-Gene Analysis

## Abstract

The Cancer Genome Atlas (TCGA) and analogous projects have yielded invaluable tumor-associated genomic data. Despite several web-based platforms designed to enhance accessibility, certain analyses require prior bioinformatic expertise. To address this need, we developed Gene ENrichment Identifier (GENI, https://www.shaullab.com/geni), which is designed to promptly compute correlations for genes of interest against the entire transcriptome and rank them against well-established biological gene sets. Additionally, it generates comprehensive tables containing genes of interest and their corresponding correlation coefficients, presented in publication-quality graphs. Furthermore, GENI has the capability to analyze multiple genes simultaneously within a given gene set, elucidating their significance within a specific biological context. Overall, GENI's user-friendly interface simplifies the biological interpretation and analysis of cancer patient-associated data, advancing the understanding of cancer biology and accelerating scientific discoveries.

## Highlights

• Analyzing the informative tumor-associated genomic databases could be challenging for non-expert users.
• GENI is a user-friendly web-based tool that provides easily discernible gene expression patterns along with biological insights.
• This website produces a table of significantly correlated genes and publication-quality multiple pathway enrichment graphs.
• GENI offers a simple-to-use tool to analyze cancer patient-derived data.

## 1 Introduction

The Cancer Genome Atlas (TCGA) [1] and similar initiatives have revolutionized cancer research by establishing extensive repositories of genomic, transcriptomic, and clinical data spanning a diverse array of cancer types [2]. These collaborative databases are pivotal resources for researchers, serving as gateways to untangle the intricate landscape of cancer and identify potential therapeutic targets. They are readily accessible through web-based platforms such as the Genomic Data Commons (GDC) (https://portal.gdc.cancer.gov/) [3] and cBioPortal (http://www.cbioportal.org/) [4], enabling investigators to examine individual genes and dissect their functions. While these web servers are of substantial value for examining user-selected genes and conducting basic analyses, a serious challenge persists for those without a strong computational background.

Here, we introduce GENI, a user-friendly, web-based platform that facilitates intuitive examination of TCGA data. GENI is designed to promptly compute correlations for genes of interest from the entire transcriptome and rank them against well-established biological gene sets, by utilizing Gene Set Enrichment Analysis (GSEA) [5]. This analytical technique assesses the behavior of entire gene sets in a certain biological context, by comparing it to predefined gene sets. The streamlined interface of GENI ensures that all users, regardless of their expertise level, can effortlessly access and analyze TCGA data, and obtain publication-quality graphs using a single tool. Furthermore, GENI possesses an additional feature, as it integrates a multigene analysis capability, enabling comprehensive investigations into cancer-associated molecular mechanisms. Together, GENI enhances the accessibility of cancer genomics databases to the diverse community of researchers.

## 2 Material & methods

**Fig. 1:**
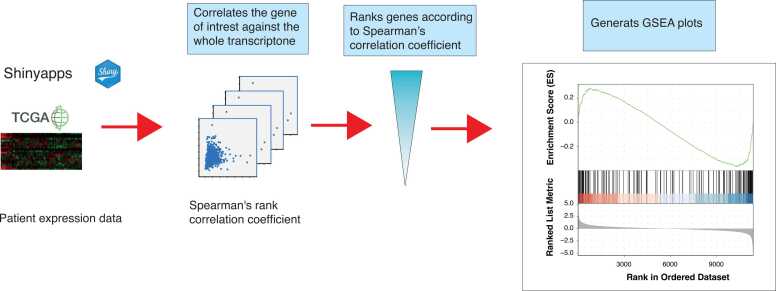
**An illustration of the GENI workflow.** Gene expression data are imported from the Shinyapps.io cloud and used to calculate Spearman's or Pearson's correlation for the gene or genes of interest against all protein-coding genes in the transcriptome. The resulting correlation values are then ranked against known gene sets. Finally, GENI produces a table of significantly correlated genes and multiple pathway enrichment graphs of publication quality.

GENI is a freely available web-based platform developed using the R programming language. It uses various packages from the open-source Bioconductor and CRAN to enable its diverse range of features. Additionally, it includes the Shiny package for efficient data analysis and visualization and the clusterProfiler for GSEA calculation [6]. Furthermore, the program calculation of Spearman’s and Pearson’s correlation coefficients is facilitated by R's built-in functions. The expression data used in GENI were obtained from multiple sources, including the TCGA website (https://dcc.icgc.org/releases/PCAWG/), NCBI (https://ftp.ncbi.nlm.nih.gov/gene/DATA/), and from the cBioPortal for Cancer Genomics which was accessed using the cBioPortalData R package [4], [7]. These diverse datasets were then combined and stored on the Shinyapps.io cloud platform (https://www.shinyapps.io). Fig. 1 provides a graphical abstract, illustrating the implementation of GENI. The complete script for GENI is accessible at https://github.com/ArataHayashi/GENI-Gene-ENrichment-Identifier.

## 3 Results

### 3.1 Workflow overview

#### 3.1.1 Single GENI

**Fig. 2:**
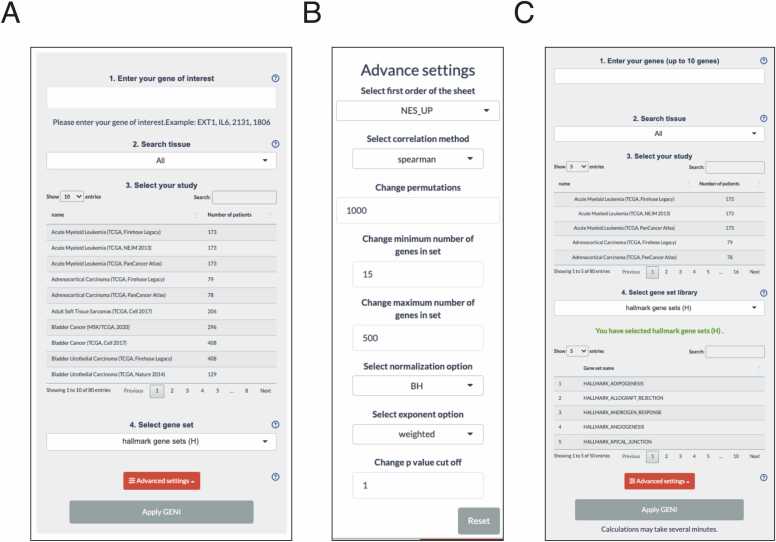
**Search panel.** (A) A screenshot of the search panel for Single GENI. (B) A screenshot of the advanced search option. (C) A screenshot of the search panel for Mutli GENI.

The GENI platform provides a streamlined search function that identifies correlations between a specific gene and the entire transcriptome using GSEA. The search begins by entering an NCBI gene ID or gene symbol in the "1. Enter your gene of interest" field (Fig. 2A). The user then selects the desired tissue and study in the "2. Search tissue" and "3. Select your study" fields, followed by choosing a gene set library obtained from the Molecular Signatures Database (MSigDB, [8]), using the droplist of "4. Select gene set" (Fig. 2A). Finally, by clicking "Apply GENI," correlation coefficients are calculated (rounded to 6 digits) and organized based on correlation values. Moreover, the "Advanced settings" button expands the search options, including the order of the summary table, correlation method, permutations, the maximum and minimum number of genes in the gene set, the p-value adjustment, the exponent, and the p-value cut-off (Fig. 2B).

The results are displayed as a table, summary dot plots, and a network plot in a new window. The user can further analyze specific gene sets of interest by clicking the indicated row in the table. This results in the appearance of a GSEA plot, enriched plot values, and a summary table of all the correlations. All of the results above can be downloaded in publication-quality PDF format and Excel format through the "Download" button.

#### 3.1.2 Multi GENI

GENI has the ability to analyze several genes simultaneously. The search begins by entering an NCBI gene ID or gene symbol in the "1. Enter your genes (up to 10 genes)" (Fig. 2C). The user selects the desired tissue and study in the "2. Search tissue" and "3. Select your study" fields. Then, the user selects a specific library and a gene set using the droplist and table found in "4. Select gene set library". Finally, by clicking "Apply GENI," Spearman's correlation coefficients are calculated (rounded to 6 digits) and organized based on correlation values. Moreover, as for a Single GENI, the "Advanced settings" button also expands the search options (Fig. 2B). The results are displayed as a table and a dot plot in a new window. The results above are presented in a high resolution and can be downloaded in PDF and Excel format through the "Download" button.

#### 3.1.3 Apply GENI to your own data

With this feature, users can upload their own data and perform GENI analysis. To ensure optimal utilization of this tool, we strongly recommend that users download the provided example file and upload their data accordingly. Once the data are uploaded, users can select the gene of interest and conduct the same analysis as in the 'Single GENI' tab. Notably, GENI can analyze multiple file formats, including txt, xlsx, tsv, and csv. Detailed instructions for data submission can be found on the main page.

#### 3.1.4 Apply GSEA

GENI provides users with the ability to upload ranked data and perform GSEA. For this functionality, users must upload a file with two columns: one containing the gene list and the other indicating the respective ranks assigned to the genes. To facilitate the analysis process, it also offers a downloadable example table in several formats and a template file for user convenience. One of GENI's advantages is its versatility in analyzing multiple file formats, including txt, xlsx, and csv.

### 3.2 Example

#### 3.2.1 Single gene analysis

To demonstrate GENI's ability to identify the biological context for specific genes, we used markers for the epithelial-mesenchymal transition (EMT) program as an example. This conserved cellular mechanism plays a significant role in cancer progression, contributing to stem-cell properties, drug resistance, immune evasion, and metastasis [9]. The induction of the EMT program is orchestrated by signaling pathways in response to extracellular cues such as the transforming growth factor-β (TGFβ) [10]. These changes include loss of cell polarity and cell-to-cell adhesion, along with alterations in the expression levels of cell surface receptors and cytoskeletal reorganization [11]. Additionally, this program induces significant changes in the cell's transcriptomic profile, as the genes associated with the mesenchymal phenotype, such as N-cadherin (CDH2), are upregulated [12]. Hence, identifying genes correlating with the EMT hallmark emerged as a promising strategy to identify unknown factors that potentially function in cancer cell aggressiveness.

**Fig. 3:**
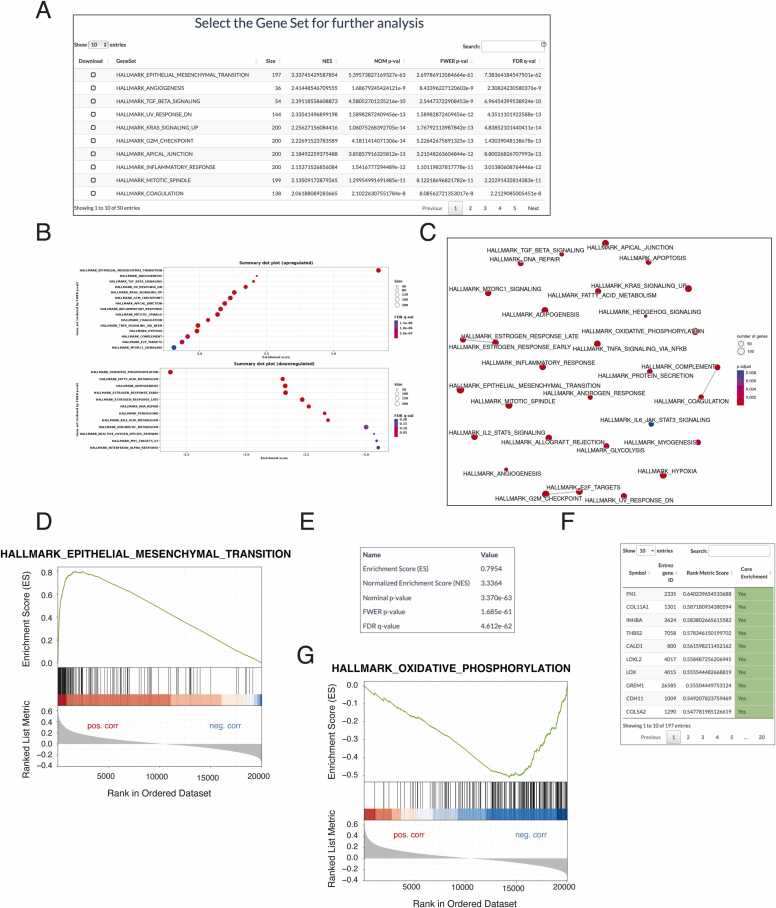
**CDH2 expression correlates with EMT markers in breast cancer patients. (**A) A screenshot of the summary table. Size: number of genes in the gene set. NES: Normalized enrichment score. NOM p-value: nominal p-value. FWER p-value: Family-wise error rate. FDRq-value: False discovery rate. (B) A screenshot of a summary dot plot of upregulated and downregulated gene sets of CDH2 correlated genes. The results are colored based on the FDR values, and the ball size is set by the number of genes in the gene set. The gene sets are sorted ascendingly according to their FWER p-values. (C) A screenshot of the network plot of CDH2 correlated gene sets. The results are colored by FDR, and the ball size is set by the gene set size. (D) GSEA plot of the Hallmark of Epithelial-Mesenchymal Transition (EMT) shows a positive and significant correlation of CDH2 with the EMT markers in breast cancer patients. (E) Screenshot of the detailed result of the hallmark of the EMT. (F) A screenshot of the EMT gene list and Spearman's rank correlation coefficient with CDH2 expression. (G) GSEA plot of Hallmark of Oxidative Phosphorylation (OXPHOS) shows a negative correlation with CDH2 expression in breast cancer patients.

In this example, we utilized GENI to search for genes co-expressed with N-cadherin in the breast invasive carcinoma (TCGA, Firehose legacy) dataset. Upon clicking the "Apply GENI" button, the intermediate table (Fig. 3A), summary plots (Fig. 3B), and network plot (Fig. 3C) of the GSEA result are displayed in the main panel. From the intermediate table, we selected the "hallmark of epithelial-mesenchymal transition" gene set as it demonstrated the highest normalized enrichment score (NES) value (Fig. 3B). Upon selecting this gene set, a GSEA plot (Fig. 3D), a detailed result table (Fig. 3E), and a gene list for the selected gene set were displayed (Fig. 3F). For comparison to the hallmark of the EMT, we selected the "hallmark of oxidative phosphorylation," as it was the gene set demonstrating the lowest NES value (Fig. 3G). Our analysis identified that N-cadherin co-expressed genes were strongly correlated with EMT, demonstrating the usefulness of GENI in identifying potential factors that function in cancer cell aggressiveness.

In a previous study utilizing GENI, we identified that the expression level of the adaptor protein dihydropyrimidines like-2 (DPYSL2) in breast cancer patients correlated with the EMT markers [13]. Next, we validated that this adaptor protein interacts with the signaling molecule Janus kinase 1 (JAK1) by applying biochemical and mice-based experimental settings. Moreover, this interaction is essential for activating the signal transducer and activator of transcription 3 (STAT3), a downstream transcription factor that regulates cancer cell aggressiveness. Hence, this study validated GENI as a tool to predict the biological function of a given gene.

#### 3.2.2 Multigene analysis

**Fig. 4:**
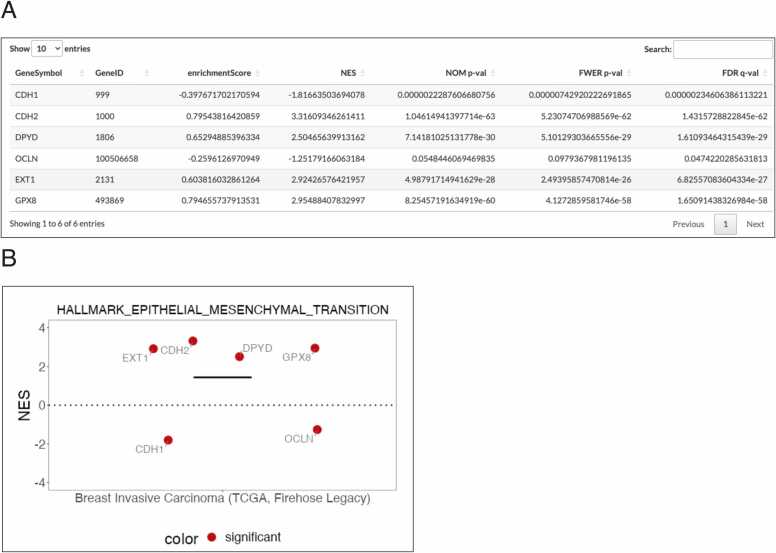
**Comparison of multi-genes by Multi GENI.** (A) A screenshot of the summary table. NES: Normalized enrichment score. NOM p-val: nominal p-value. FWER p-val: Family-wise error rate. FDR q-val: False discovery rate. (B) Dot plot of the Hallmark of Epithelial Mesenchymal Transition (EMT) on Breast invasive carcinoma (TCGA, Firehose Legacy). Colored by FDR q-value (significant: value < 0.05) NES: Normalized enrichment score.

Recent studies from our lab have highlighted the critical role of metabolic rewiring in the proper execution of the EMT program [14]. To further investigate this phenomenon, we developed a web-based tool called the Metabolic gEne RApid Visualizer (MERAV), http://merav.wi.mit.edu) [15] to systematically identify metabolic genes that are exclusively expressed in particular tumor subtypes. This analysis identified 44 metabolic genes upregulated in high-grade tumors bearing mesenchymal markers, which we designated the "mesenchymal metabolic signature" (MMS) [16]. In addition, our group confirmed three MMS genes, dihydropyrimidine dehydrogenase (DPYD), exostosin glycosyltransferase 1(EXT1) [16], and glutathione peroxide 8 (GPX8) [17], as EMT-promoting enzymes. In this example, we utilized Multi GENI to search for these two genes expressed in the breast invasive carcinoma (TCGA, Firehose legacy) dataset. We selected the Hallmark gene sets library and the "Hallmark Epithelial Mesenchymal Transition" gene set. Upon clicking the "Apply GENI" button, the summary table (Fig. 4A) and a plot (Fig. 4B) of the GSEA result were displayed in the main panel. As predicted, both genes demonstrated a significant correlation with the EMT hallmarks. In contrast, the epithelial marker occludin (OCLN) [18] presented a significant and negative NES (Fig. 4B). In addition, the expression profile of the known epithelial marker, E-cadherin (CDH1), was in contrast to the mesenchymal marker, N-cadherin (CDH2), demonstrating a clear "cadherin switch" phenomenon [9]. Therefore, GENI can distinguish between mesenchymal and epithelial genes based on the hallmark of EMT, further validating the accuracy and biological relevance of the methodology implemented in this tool.

## 4 Discussion

Various publicly available web tools are commonly utilized to ascertain the biological context of provided expression samples. Several web tools such as DAVID (https://david.ncifcrf.gov/home.jsp), Metascape (https://metascape.org), and Enricher (https://maayanlab.cloud/Enrichr/) (Table 1) analyze the expression pattern of a given and limited set of genes. Moreover, certain web tools specialize in the analysis of specific gene subsets, such as the Gene Regulatory Network Database (GRAND) (https://grand.networkmedicine.org), focusing on transcription factors, and EMTome (http://www.emtome.org) on EMT genes.

**Table 1:** **Summary table of commonly used web tools.***Gene Input:* Indicates the number of genes submitted for analysis in a single run. *Global or Selective Analysis:* Denotes the type of analysis conducted by the web tool. "Global" refers to comparing the gene of interest against the entire genome, while "Selective" indicates analysis performed exclusively on a specific set of genes. *Gene Enrichment:* Represents the type of analysis conducted by the web tool, focusing on gene enrichment. TF-transcription factors. EMT-Epithelial mesenchymal transition genes. Ref-references.

**Websites**	**Gene Input**	**Global/Selective**	**Gene Enrichment**	**Ref**
DAVID	multiple	selective	V	[19]
Metascape	multiple	selective	V	[20]
Enricher	single	selective (up to 500 genes)	V	[21]
GRAND	single/multiple (limited to TF)	selective		[22]
EMTome	single (limited to EMT)	global	V	[23]
cBioPortal	single	global		[4]
GDC	single	global		[3]
Xena	single	global		[24]
GEPIA	single	global		[25]
LinkedOmics	single	global	V	[26]
**GENI**	**single/multiple**	**global**	**V**	

Web-based platforms such as the GDC and cBioPortal have significantly impacted cancer research by providing user-friendly interfaces for researchers to access and analyze large-scale databases. Additionally, specialized bioinformatic tools such as Xena (https://xena.ucsc.edu) and Gene Expression Profiling Interactive Analysis (GEPIA) (http://gepia.cancer-pku.cn/index.html) have been developed to analyze TCGA data effectively. Specifically, these web-based tools allow the researchers to perform Kaplan-Meier survival analysis, compare tumor vs. normal tissues within or across tumors, determine the association between increased gene expression and the promoter epigenetic landscape, and create subgroups. Another notable tool is LinkedOmics (https://linkedomics.org), a web-based platform integrating multi-omics data from cancer studies, facilitating the analysis and interpretation of molecular profiles in the context of clinical outcomes. LinkedOmics provides the option to conduct various analyses such as differential expression, survival analysis, pathway enrichment, and network analysis.

Unlike these platforms with various options for analysis, GENI offers a more focused tool that results in an easy-to-use interface. This approach provides a biological context for the analysis of gene expression data and allows for the identification of potential pathways and processes involved in cancer progression. Notably, GENI has no restriction in its input, enabling the analysis of any gene in the genome. In addition, a significant advantage of GENI is its capability to analyze multiple genes simultaneously (Table 1). Moreover, the user-friendly interface of GENI makes it easy for researchers to perform complex analyses without requiring extensive bioinformatics expertise. GENI's unique feature adds another layer of analysis to this wealth of information and provides a valuable resource for the scientific community.

GENI is built on the Shinyapp platform, utilizing its capabilities to provide an intuitive and effortless interface for exploring and analyzing TCGA data through GSEA. However, it is essential to note that Shinyapp's underlying infrastructure influences the stability of the web tool. Shinyapp is a third-party service, so we cannot directly modify its structure to address stability concerns. Nevertheless, recognizing the significance of a stable user experience, we have proactively optimized our script to enhance computational efficiency within this framework. We are committed to continuously monitoring and fine-tuning GENI to ensure the best possible stability and performance, striving to provide an optimal platform for users to seamlessly engage with TCGA data and derive valuable insights into their research questions.

We have outlined forthcoming features to extend the platform's capabilities and offer a more comprehensive analytical experience. One of our main goals for the near future is to incorporate data integration capabilities with the Cancer Cell Line Encyclopedia (CCLE) and the Gene Expression Omnibus (GEO), broadening the scope of GENI's utility. These enhancements aim to enable users to conduct more comprehensive analyses by incorporating a broader range of data sources.

## 5 Conclusion

GENI provides a user-friendly and powerful web-based platform for exploring the TCGA database by allowing researchers to investigate gene expression levels relative to known gene sets. Its features are a result of its ability to conduct precise GSEA, profoundly elevating the analytical depth and biological relevance of gene expression data. Noteworthy is GENI's capability to extend the analysis beyond individual genes, which provides a broader understanding of gene interactions. Thus, GENI can potentially aid in detecting therapeutic targets and bring new perspectives to the study of cancer progression, ultimately benefiting the scientific community. Overall, GENI's importance lies in its simplicity, biological relevance, and accessibility, making it an attractive tool for cancer research.

## Funding

This work was supported by the 10.13039/501100003977Israel Science Foundation (Grant 299/21) and the Israel Cancer Research Fund project grant. AH is supported by the Brodie fellowship for breast cancer research, AH and CM by the Hebrew University International Ph.D. Talent Scholarship. MY is the Rosalind, Paul and Robin Berlin Faculty Development Chair in Perinatal Research, also supported by the Azrieli Foundation.

## Declaration of Competing Interest

The authors declare that they have no known competing financial interests or personal relationships that could have appeared to influence the work reported in this paper.
